# Crystal structure of (±)-(7*RS*,8*SR*)-7-methyl-1,4-dioxa­spiro­[4.5]decane-7,8-diol

**DOI:** 10.1107/S2056989015016783

**Published:** 2015-09-17

**Authors:** Takeshi Oishi, Hiroaki Yamamoto, Tomoya Sugai, Keisuke Fukaya, Yu Yamaguchi, Ami Watanabe, Takaaki Sato, Noritaka Chida

**Affiliations:** a School of Medicine, Keio University, Hiyoshi 4-1-1, Kohoku-ku, Yokohama 223-8521, Japan; b Department of Applied Chemistry, Faculty of Science and Technology, Keio University, Hiyoshi 3-14-1, Kohoku-ku, Yokohama 223-8522, Japan

**Keywords:** crystal structure, hydrogen bonds, paclitaxel, cyclo­hexa­ne, hy­droxy groups

## Abstract

In the title compound, C_9_H_16_O_4_, the five-membered dioxolane ring adopts a twist conformation; two adjacent C atoms deviate alternately from the mean plane of other atoms by −0.297 (4) and 0.288 (4) Å. The spiro-fused cyclo­hexane ring shows a chair form. The hy­droxy group substituted in an axial position makes an intra­molecular O—H⋯O hydrogen bond with one of the O atoms in the cyclic ether, forming an *S*(6) ring motif. In the crystal, the O—H⋯O hydrogen bond involving the equatorial hy­droxy group connects the mol­ecules into a zigzag chain with a *C*(5) motif running along the *c* axis.

## Abstract 2

In the title compound, the dioxolane and cyclo­hexane rings adopt twist and chair conformations, respectively. In the crystal, intra- and inter­molecular O—H⋯O hydrogen bonds are observed.

## Chemical context   

Paclitaxel (systematic name: (1*S*,2*S*,3*R*,4*S*,7*R*,9*S*,10*S*,12*R*,15*S*)-4,12-diacet­oxy-1,9-dihy­droxy-15-{[(2*R*,3*S*)-3-benzoylamino-2-hy­droxy-3-phen­yl]propano­yl}­oxy-10,14,17,17-tetramethyl-11-oxo-6-oxa­tetra­cyclo­[11.3.1.0^3,10^.0^4,7^]hepta­dec-13-en-2-yl benzoate) is a well-known natural diterpenoid with a potent anti­tumor activity (Wall & Wani, 1995 ▸). Its rather complicated structure and significant bioactivity have attracted chemical and medicinal inter­ests. While we recently reported several structures of the compounds (Oishi, Yamaguchi *et al.*, 2015 ▸; Oishi, Fukaya *et al.*, 2015*a* ▸,*b* ▸) obtained in the synthesis of paclitaxel (Fukaya, Tanaka *et al.*, 2015 ▸; Fukaya, Kodama *et al.*, 2015 ▸), the title compound has been prepared in an efficient synthetic approach to furnish the highly functionalized cyclo­hexane unit (Fukaya, Sugai *et al.*, 2015 ▸). Although the title compound has been reported first with a different synthetic procedure, any stereochemical or conformational assignment was not mentioned (Li *et al.*, 1981 ▸).
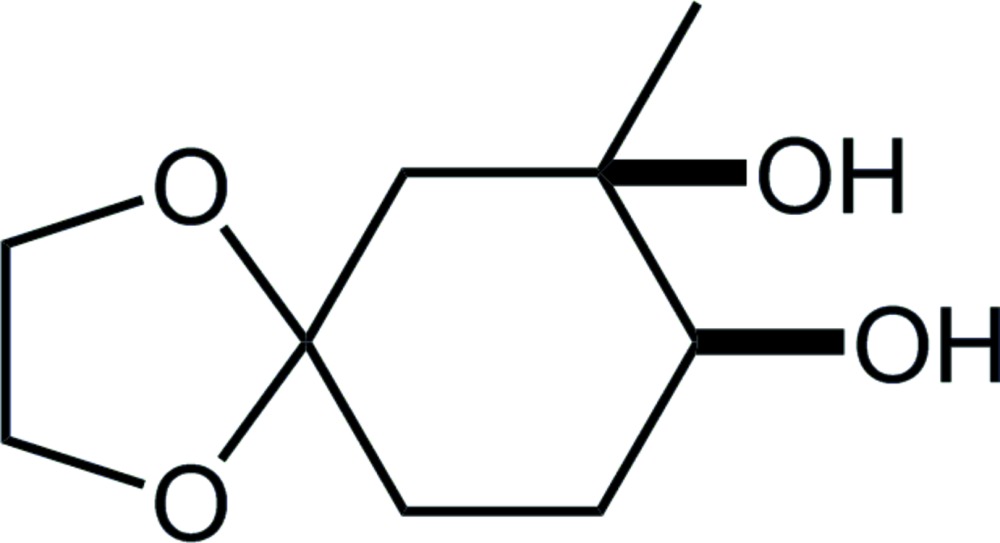


## Structural commentary   

**Figure 1:**
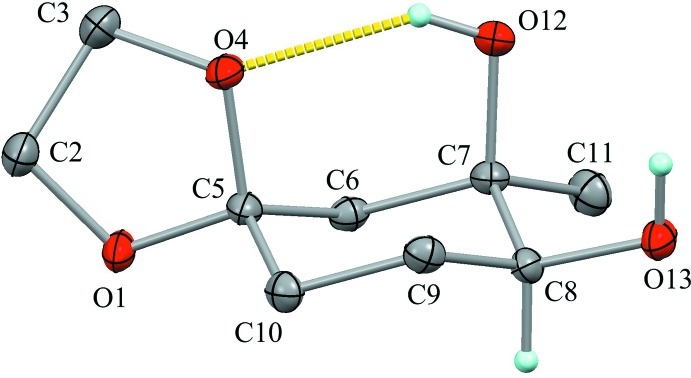
The mol­ecular structure of the title compound, showing the atom labels. Displacement ellipsoids are drawn at the 50% probability level. The yellow dotted line indicates the intra­molecular O—H⋯O hydrogen bond. Only H atoms connected to O and chiral C atoms are shown for clarity.

The mol­ecular structure of the title compound is shown in Fig. 1 ▸. The dioxolane ring (O1/C2/C3/O4/C5) adopts a twist form with puckering parameters of *Q*(2) = 0.3523 (16) Å and *φ*(2) = 233.8 (3)°. Atoms C2 and C3 deviate from the mean plane of the other three atoms by −0.297 (4) and 0.288 (4) Å, respectively. The cyclo­hexane ring (C5–C10) adopts a chair form with puckering parameters of *Q* = 0.5560 (18) Å, *θ* = 3.32 (18)°, *φ* = 193 (3)°, *Q*(2) = 0.0323 (17) Å and *Q*(3) = 0.5551 (18) Å. The C5—O1, C7—C11 and C8—O13 bonds of equatorially oriented substituents make angles of 68.30 (9), 69.85 (9) and 75.76 (9)°, respectively, with the normal to the Cremer and Pople plane of the cyclo­hexane ring. The axially oriented hy­droxy group forms an intra­molecular O—H⋯O hydrogen bond (O12—H12⋯O4; Table 1 ▸), generating an *S*(6) graph-set motif. In this ring motif, five atoms (C5—O4⋯H12—O12—O7) are nearly coplanar with a maximum deviation of 0.012 (5) Å for atom O4.

## Supra­molecular features   

**Figure 2:**
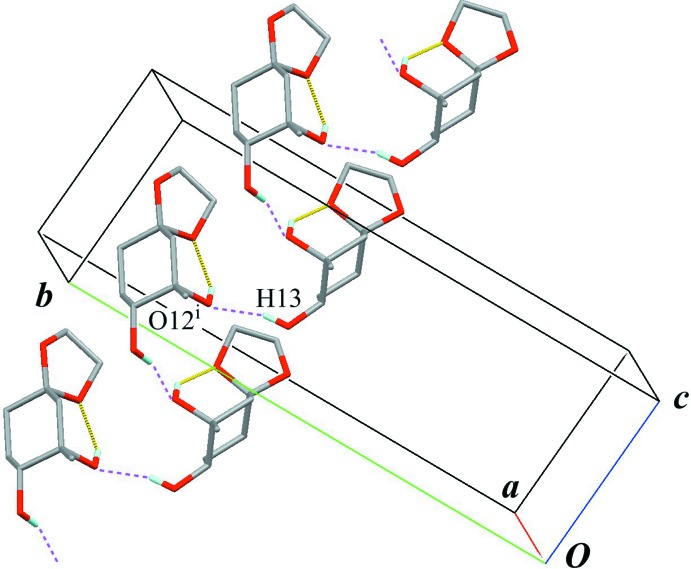
A partial packing view showing the chain structure. Yellow lines indicate the intra­molecular O—H⋯O hydrogen bonds. Purple dashed lines indicate the inter­molecular O—H⋯O hydrogen bonds. Only H atoms involved in hydrogen bonds are shown for clarity. [Symmetry code: (i) *x*, −*y* + 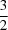, *z* − 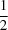.]

**Figure 3:**
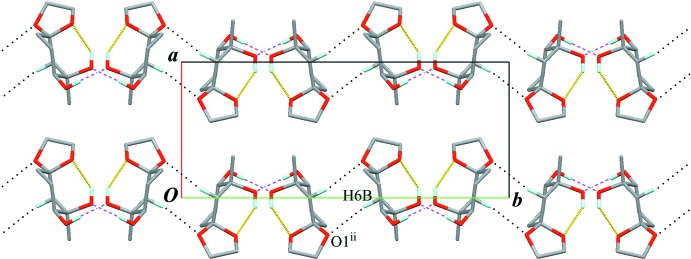
A packing diagram viewed down the *c* axis. Black dotted lines indicate the inter­molecular C—H⋯O inter­actions. Yellow lines and purple dashed lines indicate the intra- and inter­molecular O—H⋯O hydrogen bonds, respectively. Only H atoms involved in hydrogen bonds are shown for clarity. [Symmetry code: (ii) −*x*, −*y* + 1, −*z* + 2.]

The crystal packing features an inter­molecular O—H⋯O hydrogen bond (O13—H13⋯O12^i^; Table 1 ▸) connecting enanti­omers related by a glide plane to form a chain structure with a *C*(5) graph-set motif running along the *c* axis (Fig. 2 ▸). An inter­molecular C—H⋯O inter­action (C6—H6*B*⋯O1^ii^; Table 1 ▸) with a slightly longer distance, leading to form a sheet parallel to (100), is also observed (Fig. 3 ▸).

## Database survey   

**Figure 4:**
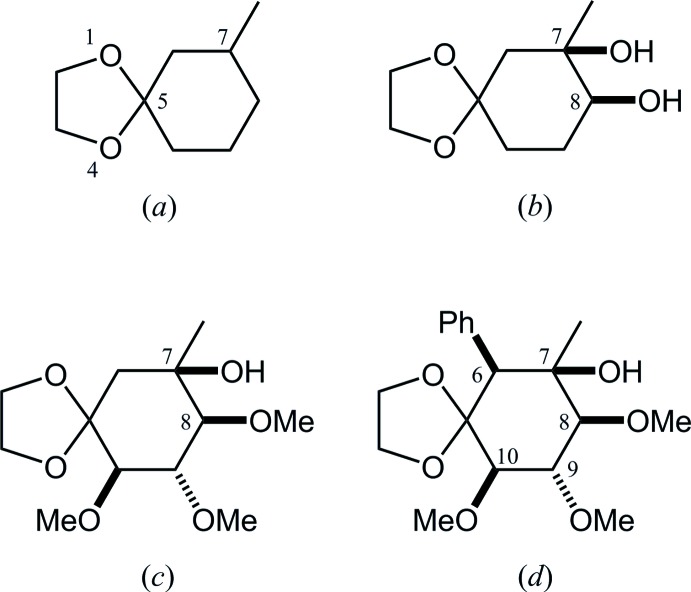
(*a*) 7-Methyl-1,4-dioxa­spiro­[4.5]decane; as the core structure for database survey, (*b*) the title compound, and its (*c*) 9,10-dimeth­oxy-8-*O*-methyl and (*d*) 9,10-dimeth­oxy-6-phenyl-8-*O*-methyl derivatives.

In the Cambridge Structural Database (CSD, Version 5.36, November 2014; Groom & Allen, 2014 ▸), 266 structures containing a 7-methyl-1,4-dioxa­spiro­[4.5]decane skeleton, (*a*), are registered (Fig. 4 ▸). These include six compounds with 7,8-di­oxy-substituents. Two of them (JIQFIY and JIQGAR; Collins *et al.*, 1998 ▸), synthesized from d-glucose, are closely related to the title compound [(*b*); racemic, *P*2_1_/*c*], which are its 9,10-dimeth­oxy-8-*O*-methyl [(*c*); chiral, *P*2_1_2_1_2_1_] and 9,10-dimeth­oxy-6-phenyl-8-*O*-methyl [(*d*); chiral, *P*2_1_2_1_2_1_] derivatives. In the crystal structures of (*c*) and (*d*), the dioxolane rings adopt twist forms and the cyclo­hexane rings show chair forms. The intra­molecular O—H⋯O hydrogen bond is also observed in (*c*), but not in (*d*).

## Synthesis and crystallization   

The title compound was afforded in an improved synthetic approach of paclitaxel from 3-methyl­anisole (Fukaya, Sugai *et al.*, 2015 ▸). Purification was carried out by silica gel column chromatography, and colorless crystals were obtained from an ethyl acetate solution by slow evaporation at ambient temperature. M.p. 359–360 K. HRMS (ESI) *m*/*z* calculated for C_9_H_16_O_4_Na^+^ [*M* + Na]^+^: 211.0946; found: 211.0936. Analysis calculated for C_9_H_16_O_4_: C 57.43, H 8.57%; found: C 57.51, H 8.50%. It is noted that the crystals grown from a diethyl ether solution under a pentane-saturated atmosphere were non-merohedral twins, and the structure is essentially the same as that reported here.

## Refinement   

Crystal data, data collection and structure refinement details are summarized in Table 2 ▸. C-bound H atoms were positioned geometrically with C—H = 0.98–1.00 Å, and constrained to ride on their parent atoms with *U*_iso_(H) = 1.2*U*_eq_(C) or 1.5*U*_eq_(methyl C). The hy­droxy H atoms were placed guided by difference maps, with O—H = 0.84 Å and with *U*_iso_(H) = 1.5*U*_eq_(O).

## Supplementary Material

Crystal structure: contains datablock(s) global, I. DOI: 10.1107/S2056989015016783/is5419sup1.cif

Structure factors: contains datablock(s) I. DOI: 10.1107/S2056989015016783/is5419Isup2.hkl

Supporting information file. DOI: 10.1107/S2056989015016783/is5419Isup3.cml

CCDC reference: 1422946

Additional supporting information:  crystallographic information; 3D view; checkCIF report

## Figures and Tables

**Table 1:** Hydrogen-bond geometry (, )

*D*H*A*	*D*H	H*A*	*D* *A*	*D*H*A*
O12H12O4	0.84	2.05	2.7838(16)	146
O13H13O12^i^	0.84	1.99	2.8093(16)	166
C6H6*B*O1^ii^	0.99	2.61	3.5631(19)	162

Symmetry codes: (i) 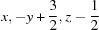; (ii) 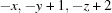.

**Table 2:** Experimental details

Crystal data	
Chemical formula	C_9_H_16_O_4_
*M*_r_	188.22
Crystal system, space group	Monoclinic, *P*2_1_/*c*
Temperature (K)	90
*a*, *b*, *c* ()	7.7403(5), 18.1498(11), 6.7335(5)
()	103.281(2)
*V* (^3^)	920.66(11)
*Z*	4
Radiation type	Mo *K*
(mm^1^)	0.11
Crystal size (mm)	0.28 0.27 0.25

Data collection	
Diffractometer	Bruker D8 Venture
Absorption correction	Multi-scan (*SADABS*; Bruker, 2014 ▸)
*T*_min_, *T*_max_	0.97, 0.97
No. of measured, independent and observed [*I* > 2(*I*)] reflections	8165, 1612, 1205
*R*_int_	0.037
(sin /)_max_ (^1^)	0.595

Refinement	
*R*[*F* ^2^ > 2(*F* ^2^)], *wR*(*F* ^2^), *S*	0.036, 0.092, 1.01
No. of reflections	1612
No. of parameters	121
H-atom treatment	H-atom parameters constrained
_max_, _min_ (e ^3^)	0.25, 0.27

Computer programs: *APEX2* and *SAINT* (Bruker, 2014 ▸), *SHELXS2013* (Sheldrick, 2008 ▸), *SHELXL2014* (Sheldrick, 2015 ▸), *Mercury* (Macrae *et al.*, 2006 ▸), *publCIF* (Westrip, 2010 ▸) and *PLATON* (Spek, 2009 ▸).

## supplementary crystallographic information

### Crystal data

**Table:**

C_9_H_16_O_4_	*D*_x_ = 1.358 Mg m^−^^3^
*M**_r_* = 188.22	Melting point: 360.2 K
Monoclinic, *P*2_1_/*c*	Mo *K*α radiation, λ = 0.71073 Å
*a* = 7.7403 (5) Å	Cell parameters from 2733 reflections
*b* = 18.1498 (11) Å	θ = 2.7–24.7°
*c* = 6.7335 (5) Å	µ = 0.11 mm^−^^1^
β = 103.281 (2)°	*T* = 90 K
*V* = 920.66 (11) Å^3^	Prism, colorless
*Z* = 4	0.28 × 0.27 × 0.25 mm
*F*(000) = 408	

### Data collection

**Table:**

Bruker D8 Venture diffractometer	1612 independent reflections
Radiation source: fine-focus sealed tube	1205 reflections with *I* > 2σ(*I*)
Multilayered confocal mirror monochromator	*R*_int_ = 0.037
Detector resolution: 10.4167 pixels mm^-1^	θ_max_ = 25.0°, θ_min_ = 2.7°
φ and ω scans	*h* = −9→8
Absorption correction: multi-scan (*SADABS*; Bruker, 2014)	*k* = −21→21
*T*_min_ = 0.97, *T*_max_ = 0.97	*l* = −8→7
8165 measured reflections	

### Refinement

**Table:**

Refinement on *F*^2^	Primary atom site location: structure-invariant direct methods
Least-squares matrix: full	Secondary atom site location: difference Fourier map
*R*[*F*^2^ > 2σ(*F*^2^)] = 0.036	Hydrogen site location: inferred from neighbouring sites
*wR*(*F*^2^) = 0.092	H-atom parameters constrained
*S* = 1.01	*w* = 1/[σ^2^(*F*_o_^2^) + (0.0407*P*)^2^ + 0.4103*P*] where *P* = (*F*_o_^2^ + 2*F*_c_^2^)/3
1612 reflections	(Δ/σ)_max_ = 0.008
121 parameters	Δρ_max_ = 0.25 e Å^−^^3^
0 restraints	Δρ_min_ = −0.27 e Å^−^^3^

### Special details

**Table:**

Experimental. IR (KBr) 3476, 3398, 2986, 2950, 2931, 2895, 1448, 1419, 1397, 1356, 1229, 1120, 1083, 1060, 1013, 952, 840, 696 cm^-1^; ^1^H NMR (500 MHz, CDCl_3_) *δ* (p.p.m.) 4.02–3.91 (m, 4H), 3.73 (s, 1H), 3.33 (ddd, *J* = 10.7, 10.6, 4.9 Hz, 1H), 2.03 (d, *J* = 10.6 Hz, 1H), 1.94–1.86 (m, 2H), 1.78–1.56 (m, 4H), 1.25 (d, *J* = 0.9 Hz, 3H); ^13^C NMR (125 MHz, CDCl_3_) *δ* (p.p.m.) 108.7 (C), 74.0 (CH), 72.5 (C), 64.7 (CH_2_), 64.4 (CH_2_), 44.1 (CH_2_), 33.2 (CH_2_), 28.4 (CH_2_), 26.2 (CH_3_).
Geometry. All e.s.d.'s (except the e.s.d. in the dihedral angle between two l.s. planes) are estimated using the full covariance matrix. The cell e.s.d.'s are taken into account individually in the estimation of e.s.d.'s in distances, angles and torsion angles; correlations between e.s.d.'s in cell parameters are only used when they are defined by crystal symmetry. An approximate (isotropic) treatment of cell e.s.d.'s is used for estimating e.s.d.'s involving l.s. planes.
Refinement. Refinement of *F*^2^ against ALL reflections. The weighted *R*-factor *wR* and goodness of fit *S* are based on *F*^2^, conventional *R*-factors *R* are based on *F*, with *F* set to zero for negative *F*^2^. The threshold expression of *F*^2^ > σ(*F*^2^) is used only for calculating *R*-factors(gt) *etc*. and is not relevant to the choice of reflections for refinement. *R*-factors based on *F*^2^ are statistically about twice as large as those based on *F*, and *R*- factors based on ALL data will be even larger.Problematic one reflection with |*I*(obs)-*I*(calc)|/σW(*I*) greater than 10 (0 2 0) has been omitted in the final refinement.

### Fractional atomic coordinates and isotropic or equivalent isotropic displacement parameters (Å^2^)

**Table:**

	*x*	*y*	*z*	*U*_iso_*/*U*_eq_	
O1	0.27151 (15)	0.55328 (6)	1.12431 (17)	0.0191 (3)	
C2	0.4342 (2)	0.58896 (9)	1.2186 (3)	0.0209 (4)	
H2A	0.5326	0.5725	1.158	0.025*	
H2B	0.4662	0.5796	1.3675	0.025*	
C3	0.3914 (2)	0.66886 (9)	1.1734 (3)	0.0204 (4)	
H3A	0.327	0.6902	1.271	0.025*	
H3B	0.5002	0.698	1.1772	0.025*	
O4	0.28135 (15)	0.66576 (6)	0.97209 (18)	0.0177 (3)	
C5	0.1875 (2)	0.59633 (9)	0.9519 (2)	0.0154 (4)	
C6	−0.0045 (2)	0.60871 (9)	0.9585 (2)	0.0139 (4)	
H6A	−0.0085	0.6365	1.0839	0.017*	
H6B	−0.0617	0.5603	0.9663	0.017*	
C7	−0.1103 (2)	0.65073 (8)	0.7741 (3)	0.0139 (4)	
C8	−0.0926 (2)	0.61203 (9)	0.5774 (2)	0.0141 (4)	
H8	−0.1427	0.5614	0.5804	0.017*	
C9	0.1009 (2)	0.60306 (9)	0.5692 (3)	0.0161 (4)	
H9A	0.1551	0.6523	0.5649	0.019*	
H9B	0.1079	0.5764	0.443	0.019*	
C10	0.2047 (2)	0.56059 (9)	0.7546 (3)	0.0160 (4)	
H10A	0.1598	0.5094	0.7496	0.019*	
H10B	0.3315	0.5586	0.7499	0.019*	
C11	−0.3033 (2)	0.65735 (10)	0.7850 (3)	0.0209 (4)	
H11A	−0.3108	0.6838	0.9097	0.031*	
H11B	−0.3544	0.608	0.7868	0.031*	
H11C	−0.3692	0.6845	0.6658	0.031*	
O12	−0.04355 (15)	0.72524 (6)	0.77185 (18)	0.0170 (3)	
H12	0.0652	0.726	0.828	0.026*	
O13	−0.19319 (15)	0.64738 (6)	0.40008 (17)	0.0179 (3)	
H13	−0.1446	0.6875	0.3825	0.027*	

### Atomic displacement parameters (Å^2^)

**Table:**

	*U*^11^	*U*^22^	*U*^33^	*U*^12^	*U*^13^	*U*^23^
O1	0.0174 (6)	0.0186 (7)	0.0174 (7)	−0.0029 (5)	−0.0040 (5)	0.0046 (5)
C2	0.0167 (9)	0.0220 (10)	0.0206 (10)	−0.0018 (8)	−0.0024 (7)	−0.0001 (8)
C3	0.0197 (10)	0.0203 (10)	0.0186 (10)	−0.0022 (8)	−0.0012 (8)	−0.0022 (8)
O4	0.0167 (6)	0.0158 (6)	0.0178 (7)	−0.0057 (5)	−0.0021 (5)	0.0019 (5)
C5	0.0157 (9)	0.0124 (8)	0.0162 (9)	−0.0029 (7)	−0.0001 (7)	0.0041 (7)
C6	0.0165 (9)	0.0130 (9)	0.0130 (9)	−0.0021 (7)	0.0048 (7)	−0.0011 (7)
C7	0.0154 (9)	0.0099 (8)	0.0166 (10)	−0.0008 (7)	0.0042 (7)	0.0003 (7)
C8	0.0160 (9)	0.0114 (9)	0.0132 (9)	−0.0010 (7)	−0.0002 (7)	0.0010 (7)
C9	0.0178 (9)	0.0162 (9)	0.0147 (9)	−0.0001 (7)	0.0045 (7)	−0.0020 (7)
C10	0.0135 (9)	0.0164 (9)	0.0188 (10)	0.0001 (7)	0.0051 (7)	−0.0005 (7)
C11	0.0181 (9)	0.0223 (10)	0.0233 (10)	0.0017 (8)	0.0071 (8)	0.0005 (8)
O12	0.0176 (6)	0.0129 (6)	0.0195 (7)	−0.0011 (5)	0.0021 (5)	−0.0007 (5)
O13	0.0192 (7)	0.0171 (6)	0.0145 (7)	−0.0021 (5)	−0.0019 (5)	0.0036 (5)

### Geometric parameters (Å, º)

**Table:**

O1—C5	1.4265 (19)	C7—C11	1.517 (2)
O1—C2	1.428 (2)	C7—C8	1.532 (2)
C2—C3	1.503 (2)	C8—O13	1.4200 (19)
C2—H2A	0.99	C8—C9	1.520 (2)
C2—H2B	0.99	C8—H8	1.0
C3—O4	1.427 (2)	C9—C10	1.529 (2)
C3—H3A	0.99	C9—H9A	0.99
C3—H3B	0.99	C9—H9B	0.99
O4—C5	1.4453 (19)	C10—H10A	0.99
C5—C10	1.512 (2)	C10—H10B	0.99
C5—C6	1.514 (2)	C11—H11A	0.98
C6—C7	1.526 (2)	C11—H11B	0.98
C6—H6A	0.99	C11—H11C	0.98
C6—H6B	0.99	O12—H12	0.84
C7—O12	1.4491 (19)	O13—H13	0.84
			
C5—O1—C2	107.70 (12)	O12—C7—C8	108.41 (13)
O1—C2—C3	102.52 (13)	C11—C7—C8	111.28 (14)
O1—C2—H2A	111.3	C6—C7—C8	109.66 (13)
C3—C2—H2A	111.3	O13—C8—C9	111.85 (13)
O1—C2—H2B	111.3	O13—C8—C7	112.27 (13)
C3—C2—H2B	111.3	C9—C8—C7	111.41 (13)
H2A—C2—H2B	109.2	O13—C8—H8	107.0
O4—C3—C2	102.13 (13)	C9—C8—H8	107.0
O4—C3—H3A	111.3	C7—C8—H8	107.0
C2—C3—H3A	111.3	C8—C9—C10	111.21 (14)
O4—C3—H3B	111.3	C8—C9—H9A	109.4
C2—C3—H3B	111.3	C10—C9—H9A	109.4
H3A—C3—H3B	109.2	C8—C9—H9B	109.4
C3—O4—C5	107.54 (12)	C10—C9—H9B	109.4
O1—C5—O4	105.99 (12)	H9A—C9—H9B	108.0
O1—C5—C10	111.37 (13)	C5—C10—C9	111.42 (13)
O4—C5—C10	108.24 (13)	C5—C10—H10A	109.3
O1—C5—C6	108.93 (13)	C9—C10—H10A	109.3
O4—C5—C6	110.10 (13)	C5—C10—H10B	109.3
C10—C5—C6	112.02 (13)	C9—C10—H10B	109.3
C5—C6—C7	113.40 (13)	H10A—C10—H10B	108.0
C5—C6—H6A	108.9	C7—C11—H11A	109.5
C7—C6—H6A	108.9	C7—C11—H11B	109.5
C5—C6—H6B	108.9	H11A—C11—H11B	109.5
C7—C6—H6B	108.9	C7—C11—H11C	109.5
H6A—C6—H6B	107.7	H11A—C11—H11C	109.5
O12—C7—C11	106.50 (13)	H11B—C11—H11C	109.5
O12—C7—C6	110.42 (13)	C7—O12—H12	109.5
C11—C7—C6	110.50 (14)	C8—O13—H13	109.5
			
C5—O1—C2—C3	30.97 (17)	C5—C6—C7—C8	53.80 (17)
O1—C2—C3—O4	−37.36 (16)	O12—C7—C8—O13	−61.28 (17)
C2—C3—O4—C5	30.55 (17)	C11—C7—C8—O13	55.53 (17)
C2—O1—C5—O4	−12.62 (16)	C6—C7—C8—O13	178.10 (12)
C2—O1—C5—C10	104.89 (15)	O12—C7—C8—C9	65.06 (16)
C2—O1—C5—C6	−131.06 (14)	C11—C7—C8—C9	−178.13 (13)
C3—O4—C5—O1	−12.23 (16)	C6—C7—C8—C9	−55.56 (17)
C3—O4—C5—C10	−131.81 (14)	O13—C8—C9—C10	−176.36 (12)
C3—O4—C5—C6	105.44 (15)	C7—C8—C9—C10	57.07 (18)
O1—C5—C6—C7	−176.78 (12)	O1—C5—C10—C9	174.98 (12)
O4—C5—C6—C7	67.39 (16)	O4—C5—C10—C9	−68.89 (16)
C10—C5—C6—C7	−53.12 (18)	C6—C5—C10—C9	52.69 (18)
C5—C6—C7—O12	−65.60 (17)	C8—C9—C10—C5	−55.09 (18)
C5—C6—C7—C11	176.83 (13)		

### Hydrogen-bond geometry (Å, º)

**Table:**

*D*—H···*A*	*D*—H	H···*A*	*D*···*A*	*D*—H···*A*
O12—H12···O4	0.84	2.05	2.7838 (16)	146
O13—H13···O12^i^	0.84	1.99	2.8093 (16)	166
C6—H6*B*···O1^ii^	0.99	2.61	3.5631 (19)	162

Symmetry codes: (i) *x*, −*y*+3/2, *z*−1/2; (ii) −*x*, −*y*+1, −*z*+2.

## References

[bb1] Bruker (2014). *APEX2*, *SAINT* and *SADABS*. Bruker AXS Inc., Madison, Wisconsin, USA.

[bb2] Collins, D. J., Hibberd, A. I., Skelton, B. W. & White, A. H. (1998). *Aust. J. Chem.* **51**, 681–694.

[bb3] Fukaya, K., Kodama, K., Tanaka, Y., Yamazaki, H., Sugai, T., Yamaguchi, Y., Watanabe, A., Oishi, T., Sato, T. & Chida, N. (2015). *Org. Lett.* **17**, 2574–2577. 10.1021/acs.orglett.5b01174 26010999

[bb4] Fukaya, K., Sugai, T., Sugai, T., Yamaguchi, Y., Watanabe, A., Yamamoto, H., Sato, T. & Chida, N. (2015). In preparation.

[bb5] Fukaya, K., Tanaka, Y., Sato, A. C., Kodama, K., Yamazaki, H., Ishimoto, T., Nozaki, Y., Iwaki, Y. M., Yuki, Y., Umei, K., Sugai, T., Yamaguchi, Y., Watanabe, A., Oishi, T., Sato, T. & Chida, N. (2015). *Org. Lett.* **17**, 2570–2573. 10.1021/acs.orglett.5b01173 26010812

[bb6] Groom, C. R. & Allen, F. H. (2014). *Angew. Chem. Int. Ed.* **53**, 662–671. 10.1002/anie.201306438 24382699

[bb7] Li, Y.-L., Pan, X.-F., Huang, W.-K., Wang, Y.-K. & Li, Y.-C. (1981). *Acta Chim. Sin.* **39**, 937–939.

[bb8] Macrae, C. F., Edgington, P. R., McCabe, P., Pidcock, E., Shields, G. P., Taylor, R., Towler, M. & van de Streek, J. (2006). *J. Appl. Cryst.* **39**, 453–457.

[bb9] Oishi, T., Fukaya, K., Yamaguchi, Y., Sugai, T., Watanabe, A., Sato, T. & Chida, N. (2015*a*). *Acta Cryst.* E**71**, 466–472. 10.1107/S2056989015006854 PMC4420046 25995857

[bb10] Oishi, T., Fukaya, K., Yamaguchi, Y., Sugai, T., Watanabe, A., Sato, T. & Chida, N. (2015*b*). *Acta Cryst.* E**71**, 490–493. 10.1107/S2056989015007136 PMC4420122 25995863

[bb11] Oishi, T., Yamaguchi, Y., Fukaya, K., Sugai, T., Watanabe, A., Sato, T. & Chida, N. (2015). *Acta Cryst.* E**71**, 8–11. 10.1107/S2056989014026048 PMC4331859 25705437

[bb12] Sheldrick, G. M. (2008). *Acta Cryst.* A**64**, 112–122. 10.1107/S0108767307043930 18156677

[bb16] Sheldrick, G. M. (2015). *Acta Cryst.* C**71**, 3–8.

[bb13] Spek, A. L. (2009). *Acta Cryst.* D**65**, 148–155. 10.1107/S090744490804362X PMC2631630 19171970

[bb14] Wall, M. E. & Wani, M. C. (1995). *ACS Symp. Ser.* **583**, 18–30.

[bb15] Westrip, S. P. (2010). *J. Appl. Cryst.* **43**, 920–925.

